# Development of Multiple Capsule Robots in Pipe

**DOI:** 10.3390/mi9060259

**Published:** 2018-05-25

**Authors:** Shuxiang Guo, Qiuxia Yang, Luchang Bai, Yan Zhao

**Affiliations:** 1 The Institute of Advanced Biomedical Engineering System, School of Life Science, Beijing Institute of Technology, No. 5, Zhongguancun South Street, Haidian District, Beijing 100081, China; guo@eng.kagawa-u.ac.jp (S.G.); blc1223615376@bit.edu.cn (L.B.); zhaoyan@bit.edu.cn (Y.Z.); 2 Key Laboratory of Convergence Medical Engineering System and Healthcare Technology, Ministry of Industry and Information Technology, Beijing Institute of Technology, Beijing 100081, China; 3 Intelligent Mechanical Systems Engineering Department, Kagawa University, Takamatsu 761-0396, Japan

**Keywords:** multiple capsule robots, rotational electromagnetic field, screw structure, docking and release

## Abstract

Swallowable capsule robots which travel in body cavities to implement drug delivery, minimally invasive surgery, and diagnosis have provided great potential for medical applications. However, the space constraints of the internal environment and the size limitations of the robots are great challenges to practical application. To address the fundamental challenges of narrow body cavities, a different-frequency driven approach for multiple capsule robots with screw structure manipulated by external electromagnetic field is proposed in this paper. The multiple capsule robots are composed of driven permanent magnets, joint permanent magnets, and a screw body. The screw body generates a propulsive force in a fluidic environment. Moreover, robots can form new constructions via mutual docking and release. To provide manipulation guidelines for active locomotion, a dynamic model of axial propulsion and circumferential torque is established. The multiple start and step-out frequencies for multiple robots are defined theoretically. Moreover, the different-frequency driven approach based on geometrical parameters of screw structure and the overlap angles of magnetic polarities is proposed to drive multiple robots in an identical electromagnetic field. Finally, two capsule robots were prototyped and experiments in a narrow pipe were conducted to verify the different motions such as docking, release, and cooperative locomotion. The experimental results demonstrated the validity of the driven approach for multiple capsule robots in narrow body cavities.

## 1. Introduction

Capsule robots are swallowable, untethered, mobile microrobots employed in minimally- or non-invasive procedures, which provide a promising method for precision medicine. Conventional tools, such as endoscopes, lead to blind spots due to the convoluted nature of body cavities, which are beyond the position of their reach. These treatments, either oral or rectal, rub against the cavities’ surfaces through push and pull maneuvers, and patients bear extreme discomfort during these procedures [1]. However, through only the simple and non-invasive swallowing of a pill and no anaesthesia, modern capsule robots offer an appealing alternative to traditional flexible endoscopy in the gastrointestinal tract (GI) [2]. These capsule robots are able to reach narrow regions, such as the small intestine, which is not possible with conventional endoscopes. Their essentially non-invasive nature allows for less painful diagnosis. It is widely agreed that these robots will make healthcare more portable and personal. The development of capsule robots has significant potential to revolutionize treatment procedures involving different applications. For example, drugs loaded in these robots can be delivered to target lesions [3], and some minimally invasive surgeries are able to cure diseases by simplified medical apparatus and instruments. Furthermore, the diagnostic real time data is collected and transferred out of the body for surgeons [4].

Owing to the great potential of capsule robots, mechanisms and locomotion have been studied for decades. The earthworm-like motion, inspired by biology, has been applied to capsule robots. Kim et al. [5,6] have developed two prototypes; these devices propel themselves by cyclic compression/extension of shape memory alloy (SMA) spring actuators. However, intrinsic cycle time and power consumption limits of SMA actuators limit actuation efficiency and speed. A “paddling” technique where leg-like fins travel the length of the capsule is proposed by Park et al. [7] to produce locomotion in the GI tract, where the fins retract before recycling to the front of the capsule for the next paddle stroke. This motion can obtain rapid velocity, but it cannot achieve bidirectional motion in this form. Valdastri et al. [8,9] developed a 12-legged capsule robot which performed fully bidirectional locomotion. This design was able to distend tissues in a uniform manner with six points of contact at each end of the capsule. A main challenge is the consideration of foot geometry for safe contact with the intestinal wall. Yim et al. [10] proposed a magnetically actuated soft capsule endoscope as a tetherless miniature mobile robot platform for diagnostic and therapeutic medical applications inside the stomach. The distance change between the robot and permanent magnet should be compensated in real-time for further development. 

A promising new approach for locomotion in the laminar regime is propeller or screw propulsion, where actuated by external magnetic field, the robots rotate inside the body tissues. Lee et al. [11] proposed an untethered flexible-legged magnetic robot to generate effective locomotion and precision unclogging motion. The control method based on frequency has been verified by in vitro experiments. Fu and colleagues [12] proposed a capsule robot with shrouded propeller and screw grooves rotating in a magnetic field to achieve effective propulsive performance. Moreover, the results demonstrated that the propulsive force of a shrouded propeller was larger than a bare propeller. Yu and Kim [13] designed a robotic guidewire, which was controlled by an external rotating magnetic field. The active locomotion, steering, and towing of the guidewire and drilling was verified in a silicone oil and agar jelly. Temel et al. [14,15] set a computational dynamic model of untethered robots in a channel and validated it with experiments. They provided valuable insights for the design of capsule robots with geometrical parameters. To reduce fluid distorting effect, a petal-shaped capsule robot was proposed by Zhang et al. [16,17]. The twist impact on the GI tract by the petal-shaped capsule robot was reduced, while the non-contact driving performance in the GI tract was improved greatly isolated by fluid membrane with high dynamic pressure. Furthermore, the propulsion and swimming speed of the innovative, variable-diameter capsule robot, with radial clearance compensation based on multiple wedge effects, were significantly improved [18]. Because of their self-propulsion and battery-free properties, capsule robots with screw structures driven by external magnetic fields are designed in this paper.

Although mechanisms and control strategies have achieved huge success, as mentioned above, and commercial capsule endoscopes are available for patients more easily, there are still some shortages for further clinical applications. On the one hand, due to the intrinsic size limitation of the swallowable capsules, an individual robot can hardly carry enough sensors and power units, which results in a lack of multifunction. Thus, commercial capsule robots are able to replace endoscopes, but have not been competent in biopsy or surgery. On the other hand, limited by space constraints of the GI tract, the poor dexterity of a simplex structure is another limitation for clinical application, as capsule robots lack the ability to interact with GI tissue [19].

Multiple robots technology may be able to address the single function problem. Multiple robots, also called assembling reconfigurable endoluminal surgical systems (ARES) in some cases [20], is a set of robots with different features which can realize multifunction after assembling together. A typical multiple robots set may include several robots carrying different sensors, tools or drugs. By tracing, assembly, resolution, and other motions, these multiple units detect the environments and accomplish complex manipulations. Kim et al. [21] introduced a prototype capsule system which was designed to distribute functional burdens. These robots achieved active locomotion via a collaborative actuation. Moreover, inductive transmission techniques were used to supply power. To steer robots for endoluminal surgery, Harada et al. [22,23] proposed a master device, which enables surgeons to customize the surgical system. These preliminary studies have respective shortages. The robots driven by electric energy lack effective power supply units, and these robots have not been tested in liquid environments. Nagy et al. [24,25] proposed the use magnets in a specific configuration on the mating faces of the module. Their results showed that high success rates can be achieved and snake-type robots can adapt to irregular paths. The probability of correct alignment needs to be improved. Guo et al. [26] proposed wireless spiral capsule robots with modular structures. Driven by electromagnetic fields, guide and auxiliary robots combine and separate via docking mechanisms. However, it is hard for magnetic fields to steer individual robots independently, because the drive frequencies of individual robots have the same range (i.e., one robot is out of control as the operator guides the other one, and they can only move in opposite directions). Zhang et al. [27] studied the start-up curves of different robots and employed genetic algorithms to optimize screw structures to drive several capsule robots. However, the cooperative locomotion of multiple robots has not been implemented in the real world.

In this paper, a different-frequency driven approach for multiple capsule robots in narrow tissues of the GI tract is proposed. With an enough non-overlapping range of critical driven frequencies, multiple capsule robots can move independently as well as cooperatively under an identical electromagnetic field. Two capsule robots with screw structures and docking mechanisms are designed and fabricated. Helmholtz coils generate an electromagnetic field to steer these robots to implement linear motions. The dynamic model of the capsule robot is established and the multiple start and step-out frequencies are defined theoretically. The effectiveness of docking, release, and cooperative locomotion is validated by a series of experiments in pipe. The start frequencies of the two capsule robots have an interval of 5 Hz, and the step-out frequencies of the two robots are 19 Hz and 8 Hz, separately. The axial speeds of an individual robot peak at 4.75 mm/s and 6.35 mm/s, separately. The maximum of cooperative locomotion speed achieved is 3.32 mm/s.

This paper is organized as follows. In Section 2, the mechanism and fabrication of multiple capsule robots and electromagnetic field design with Helmholtz coils are presented. In Section 3, the different-frequency driven approach to steer multiple robots under an identical rotational electromagnetic field is presented, where definition of multiple start and step-out frequencies are given theoretically based on the dynamic model. The experiments and results of different motions such as docking, release, and cooperative locomotion in pipe are described in Section 4. Finally, the conclusions and future work are summarized in Section 5.

## 2. Locomotion Mechanism and Magnetic Field Design

### 2.1. Concept and Application Procedure

**Figure 1:**
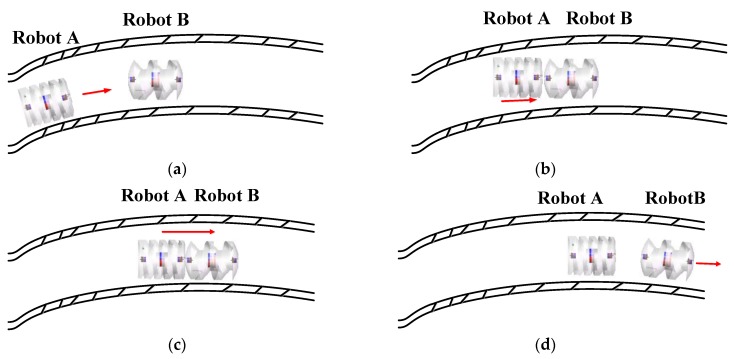
The concept of proposed multiple capsule robots in narrow body cavities: (**a**) the individual locomotion; (**b**) the docking procedure; (**c**) the cooperative locomotion; and (**d**) the release procedure.

Multiple capsule robots are proposed for GI diagnosis and surgeries. Distinguished from conventional capsule endoscopes, this set of robots contain various capsule robots with different functions determined by their respective structures and loadings. In a practical case of clinical application, the treatment procedure which involves the following steps is shown in Figure 1.

Step 1: Patients drink enough medicinal liquid prior to the deglutition of capsule robots [22,23]. That is to say, these robots have acquired enough liquid for them to travel through inner cavities and the esophagus becomes smoother as well. Afterwards, patients ingest the necessary capsule robots and surgeons guide them to the target position.

Step 2: Surgeons steer these capsule robots to dock together for a complex structure in a proper order. By assembly, reorganization, transformation, and other actions under guidance, these robots complete treatments such as drug delivery, targeted therapy, and gastric biopsy.

Step 3: At the end of the treatment, the whole construction dissolves and the multiple capsule robots are isolated from each other. The capsule robots move through the large intestine and are excreted at anal orifice.

During the treatment procedure, surgeons operate the console to generate a rotational electromagnetic field, which enables the robots to perform active locomotion. The robots also send back information on the human body and their positions within. Bidirectional data between robots and surgeons is transmitted via a wireless transmission module.

### 2.2. Structure and Materials of Multiple Capsule Robots

**Figure 2:**
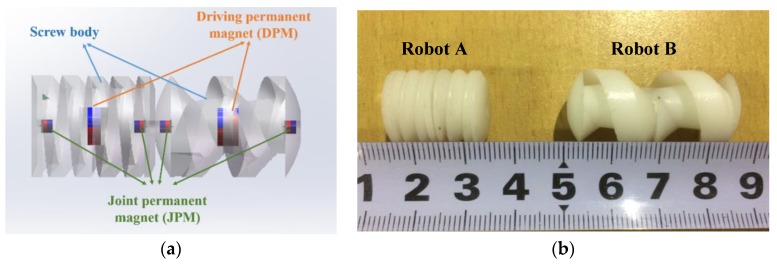
Design of multiple capsule robots: (**a**) schematics of multiple capsule robots; (**b**) prototype of multiple capsule robots.

Each capsule robot is composed of three parts: screw body, the driving permanent magnets (DPMs), and the joint permanent magnets (JPMs). In this paper, two types of capsule robots, Robot A and Robot B, are designed. The structure of the two robots is shown in Figure 2.

**Figure 3:**
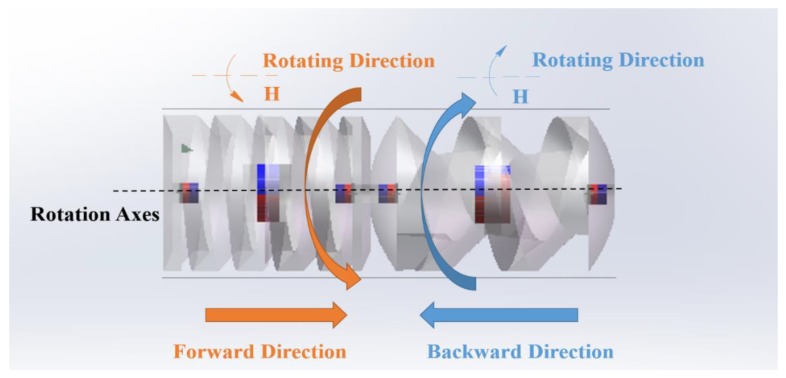
Motion mechanism of the active locomotion.

The screw body, made of resin (density: 1.2 g/cm^3^, hardness: 79 HD, tensile strength: 35 MPa, Poisson’s ratio: 0.41, water absorption: 0.4%), was fabricated using a 3D prototyping printer (UnionTech, Lite HD, Shanghai, China). It is hollow in structure in order to assemble sensors for further development, as well as to obtain large buoyancy. The water between the spiral blades generates propulsion as the multiple capsule robots rotate axially. The spirals on each robotic body have the same direction, hence the motion direction is constrained by rotation direction [28]. The active locomotion steered by rotational electromagnetic field is shown in Figure 3.

**Figure 4:**
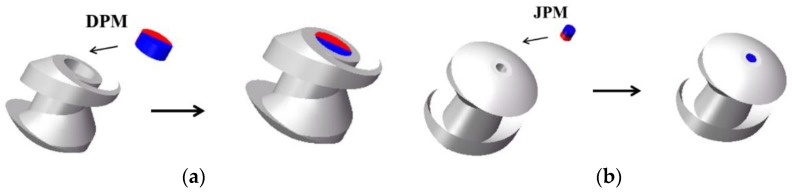
Fabrication of the capsule robots: (**a**) fabrication of DPMs; (**b**) fabrication of JPMs.

The robotic body assembles two types of permanent magnets: the driving permanent magnets (DPMs) in the middle of the body and the joint permanent magnets (JPMs) on both sides. Both of the DPMs and JPMs are made of NdFeB magnets. The DPMs are magnetized radially and JPMs are magnetized axially. The surface of each magnet was processed by nickel plating. Perpendicular to the long axis, the screw body can be split into two parts. DPMs are embedded in the middle of the robot. Meanwhile, JPMs are embedded at the two ends of the robotic body. The sizes of the grooves were designed to lock the permanent magnets. In addition, solid gum was used to conglutinate the two parts together. The fabrication diagram is shown in Figure 4. Magnetized radially, the DPMs try to follow the rotational electromagnetic field synchronously, which can generate rotating torque on the multiple capsule robots [29,30]. The docking mechanism contains two JPMs, which are placed at the two ends of the robotic body. When two capsule robots approach each other, JPMs provide attractive force and they dock in succession. Table 1 summarizes the design parameters of the two capsule robots.

### 2.3. Design of the Rotational Electromagnetic Field

It was a great challenge to provide energy to such small-scale robots because they can hardly carry sufficient batteries due to space limitations. Once these capsule robots are detained in the human body, they cannot only affect diagnosis. but also risk life. Magnetic field is a promising method for energy supply. In this paper, two pairs of Helmholtz coils are utilized to drive multiple capsule robots for linear motion.

**Figure 5:**
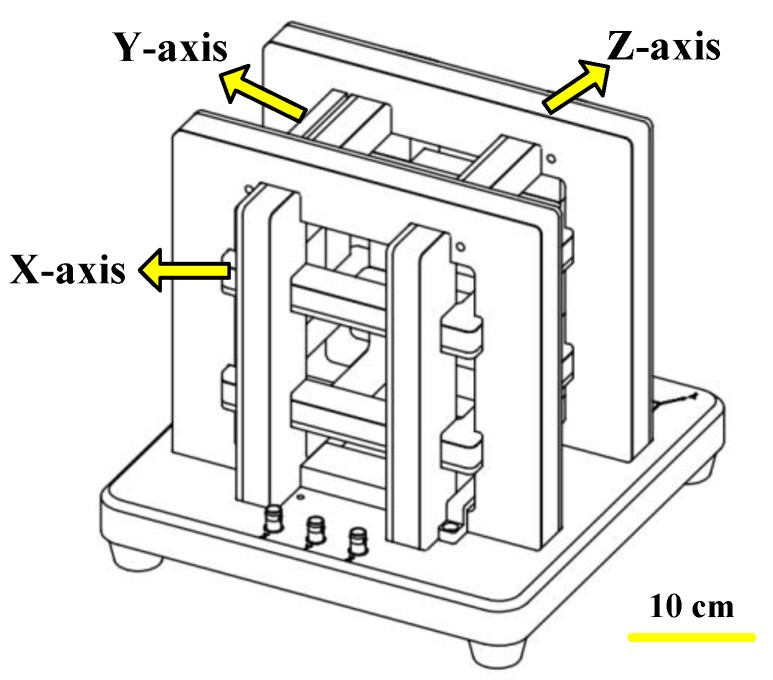
Schematics of the three-axis Helmholtz coils.

The three-axis Helmholtz coils have three pairs of symmetric coils, with flowing identical electric currents. Some of the parameters of the coils are shown in Table 2. Indeed, the electric currents generated a uniform magnetic field in the middle of the space. By adjusting phases and magnitude of current flows, the multiple capsule robots were able to perform active locomotion under the guidance of manipulators [31,32]. Figure 5 shows the schematic designs and the coordinate system set in the coils.

The capsule robots could perform linear motion with two pairs of Helmholtz coils. The electric currents flows in the X- and Z-axis coils generated a rotational magnetic field plane perpendicular to the moving direction of the capsule robots. In each pair of coils, the electric currents were identical, while the electric currents of the two axes had phase difference.

## 3. Different-Frequency Driven Approach

As is stated above, the screw structure outside the robotic body is significant for hydrodynamic analysis. In order to research the kinematic performance of the multiple capsule robots, a dynamic model based on geometrical parameters was set. Furthermore, the driven approach was also developed for these robots to start or stop by different frequencies.

Due to the focus on medical application, water was substituted for body fluids at the experimental stage. The Reynolds number—a dimensionless number used to characterize the fluid flow—determines the resistance of an object in liquid. The definition of a Reynolds number is:

(1) $$Re=\frac{\rho ul}{\mu }$$

where ρ and μ are density and viscosity of the liquid, and u and l are velocity and length scale of the flow. In this paper, the capsule robots moved in a pipe filled with water (viscosity coefficient $1.005\times 10^{-3}$ Pa⋅s). Since the practical axial speeds of the capsule robots were about 1.3 mm/s to 6.4 mm/s, and the diameter of the robots was 16 mm, the minimal and maximal Reynold numbers were 20.3 and 99.9. The environment was the laminar regime and the flow was Newtonian fluid flow.

According to Newton Viscous Law, the viscous resistance is defined by:

(2) $$f_{c}=\mu A\frac{v_{c}}{l}$$

where $f_{c}$ is the circumferential viscous resistance generated by the rotation motion. A is the area of the robot with relative motion, and $\frac{v_{c}}{l}$ is the circumferential velocity gradient [33].

**Figure 6:**
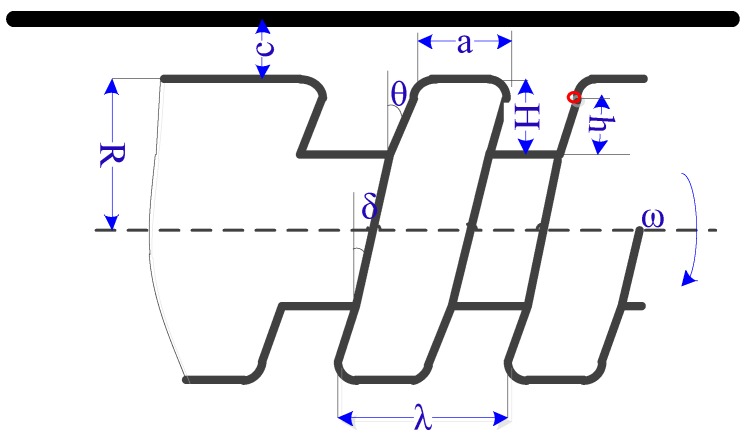
Geometrical parameters of the screw structure.

The infinitesimal method (i.e., decomposing the entity into small pitches) increased the efficiency for mechanic solution. The geometrical parameters of the capsule robot are shown in Figure 6. Picked as an infinitesimal element on the screw blade, the circumferential viscous resistance from the liquid were evaluated by the following equations:

(3) $$df_{c1}=\mu dA_{s}\frac{v_{c}}{l_{1}}$$

(4) $$df_{c2}=\mu dA_{s}\frac{v_{c}}{l_{2}}$$

where $f_{c1}$ and $f_{c2}$ are circumferential viscous resistance of left and right screw blade respectively. $dA_{s}$ is the area of the infinitesimal element’s trajectory when the capsule robot rotates one turn, $l_{1}$ is the distance between the infinitesimal element on the left blade and the pipe, and $l_{2}$ is the distance between the infinitesimal element on the right blade and the long axis. The two distances are expressed as:

(5) $$l_{1}=R+c-(R-H+h)$$

(6) $$l_{2}=R-H+h$$

where R is the radius of the capsule robot, H is the depth of the screw, and h is the radial distance between the infinitesimal element and the root of the screw. The area of the trajectory is expressed as:

(7) $$dA_{s}=Cds$$

where the ds is the width of the infinitesimal element and C is the perimeter of the trajectory. The ds is expressed as

(8) ds=dh/tanθ

where dh is the height of the infinitesimal element and θ is the blade angle. The perimeter of trajectory C is presented as:

(9) $$C=\frac{2\pi \left(R-H+h\right)}{\cos\delta }$$

where δ is the lead angle. Furthermore, the circumferential viscous resistances acting on the crest and root of the screw are expressed as:

(10) $$f_{c3}=\mu a\frac{2\pi R}{\cos\delta }\cdot \frac{\omega R}{c}$$

(11) $$f_{c4}=\mu (\lambda -a)\frac{R-H}{\cos\delta }\cdot \frac{\omega (R-H)}{H+c}$$

(12) ω=2πf

where a is the width of the screw, ω is the angular velocity of the capsule robot, f is the rotational frequency of the robot, and λ is the pitch of the screw. The total circumferential viscous resistance $f_{c}$ and torque $M_{c}$ of the capsule robot are expressed as:

(13) $$f_{c}=n(\int_{0}^{H}df_{c1}+\int_{0}^{H}df_{c2}+f_{c3}+f_{c4})$$

(14) $$M_{c}=n(\int_{0}^{H}(R-H+h)df_{c1}+\int_{0}^{H}\left(R-H+h)df_{c2}+f_{c3}R+f_{c4}(R-H\right))$$

where n is the number of screws.

In Figure 6, the circumferential viscous resistance of screw blade $f_{c12}$ perpendicular to the paper is expressed as:

(15) $$f_{c12}=n(\int_{0}^{H}df_{c1}+\int_{0}^{H}df_{c2})$$

which can also be expressed as:

(16) $$f_{c12}=\frac{f_{a}}{\tan\delta }$$

where $f_{a}$ is the propulsion along the axis.

Multiple start frequency: At low frequencies, the DPMs try to rotate synchronously with rotational electromagnetic field. Rotating at a certain velocity, the capsule robot obtains an axial force while the water flows backward. The capsule robot begins to move axially only when it can overcome friction force between the pipe and the robot. For laminar flow, friction force can be calculated as:

(17) $$F_{f}=\eta \left(G-F_{b}\right)$$

where G and $F_{b}$ are the gravity and buoyancy of the robot, η is the friction coefficient, and $F_{b}$ is given as:

(18) $$F_{b}=\rho Vg$$

where V is the volume of the capsule robot.

At low driven frequencies, the capsule robot rotates to keep pace with rotational electromagnetic field. The rotational frequencies of the robot are close to the rotation frequencies of external electromagnetic field. A capsule robot is able to move axially, only when the propulsion is greater than friction force, otherwise, the capsule robot rotates at a certain angular velocity, but remains at the initial position. With the rotational frequencies increasing, the propulsion becomes larger than the friction force. Then the robot starts the axial movement. Based on this point, the rotational frequency of a capsule robot which enables it to start axial movement is defined as “start frequency”. In this paper, the start frequency of multiple capsule robots is defined as:

(19) $$f_{start}=\left\{\begin{array}{cccc} f_{A-start} & f_{B-start} & f_{C-start} & \ldots \end{array}\right\}$$

where $f_{A-start}$, $f_{B-start}$, $f_{C-start}$ represent the start frequency of an individual robot.

Propulsion is determined by the geometrical parameters of the screw. Once the geometrical parameters are set, the start frequency is invariable. This equation shows that the propulsion is related to screw pitch, the numbers of turns, radium of the robot, the depth and width of screws, the blade angle, and the lead angle. Two robots with different start frequencies were obtained by designing these parameters. Therefore, one can move forward or backward, while the other stays in the initial position in the identical electromagnetic field.

Multiple step-out frequency: Once the capsule robot moves forward, the drag force increases as the axial speed increases. The drag force of a cylinder is defined as:

(20) $$F_{d}=\frac{1}{2}\rho C_{d}Sv^{2}$$

where S is the maximum cross area that is vertical to the flow of fluid and $C_{d}$ is the resistance coefficient. 

The rotational frequencies of the robot increase as the frequencies of the rotational electromagnetic field increase. The propulsion becomes larger which enables the robot to accelerate axially. The capsule robot keeps a constant speed when the propulsion is equal to the resultant force of friction force and drag force. Indeed, the maximum of axial speed is determined by the peak of rotation speed. Until the robot is not able to maintain synchronous rotation with external electromagnetic field, the axial speed of the capsule robot declines to zero rapidly. The rotational frequency of the robot which cannot hold synchronous rotation with external electromagnetic field is defined as “step-out frequency”. In this paper, the step-out frequency of multiple robots is defined as:

(21) $$f_{step-out}=\left\{\begin{array}{cccc} f_{A-step-out} & f_{B-step-out} & f_{C-step-out} & \ldots \end{array}\right\}$$

where $f_{A-step-out}$, $f_{B-step-out}$, $f_{C-step-out}$ represent the step-out frequency of an individual robot.

The rotation motion is controlled by the DPMs in the electromagnetic field. The magnetic force and magnetic torque of an individual DPM are given by:

(22) $$F_{m}=V_{m}\left(M\cdot \nabla \right)\times B$$

(23) $$M_{m}=V_{m}M\times B$$

where $V_{m}$ and M are the volume and magnetization of the magnet and B is the magnetic flux density.

**Figure 7:**
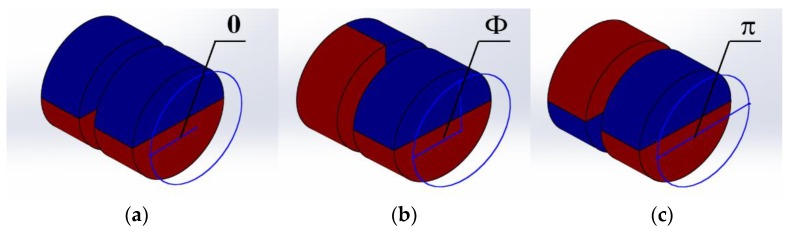
Overlap angles of two polarities: (**a**) 0 degree; (**b**) Φ degree; and (**c**) π degree.

In a certain rotational electromagnetic field, the magnetic torque is affected by DPMs. Our approach was to change the practical torque by overlapping the DPMs with different overlap angles of two polarities. The torque is offset if the opposite polarities overlap and the torque increases as the overlap of same polarities enlarges. If the overlap angle of the same polarities is 0 degree, the torque of the magnetic is twice that of an individual magnet. The step-out frequency of the robot increases. However, if the opposite polarities overlap, the torque will be offset. The robot would not rotate with the external electromagnetic field, as well as move forward. The description is shown in Figure 7. The practical torque is given by:

(24) $$M_{r}=\frac{2\left(\pi -\Phi \right)}{\pi }M_{m}$$

where Φ is the overlap angle of the opposite polarities.

According to Equation (14), at a certain rotational frequency f, the robot is able to rotate synchronously with the rotational electromagnetic field when $M_{r}\geq M_{c}$, otherwise the robot cannot maintain synchronous rotation and comes to a stop. 

Based on the dynamic model, the axial speed of a capsule robot is expressed as:

(25) $$v_{axial-1}=F\left(f,\;\lambda ,\;R,\;\Phi \cdots \right)$$

where F(f, λ, R, Φ⋯) is the function between axial speeds and variables such as driven frequencies and design parameters. However, some practical factors, such as vibration during rotation, collision with the pipe, and the interaction of multiple robots are not considered in this equation. So a correction factor is introduced to this model. The axial speed is then expressed as:

(26) $$v_{axial}=\varepsilon F\left(f,\;\lambda ,\;R,\;\Phi \cdots \right)$$

where ε is the correction factor. Verified by some experiments, ε was set at 0.5 in this paper.

The multiple capsule robots can be controlled independently under the different frequency ranges, as long as the frequency ranges have non-overlapping areas. The multiple start frequency and step-out frequency can be designed by the geometrical parameters of the robot and the overlap angles of DPMs.

## 4. Experiments and Results

### 4.1. Advance Locomotion Test of Individual Robots

According to Section 3, the geometrical parameters and overlap angle of magnetic polarities were designed separately. The parameters are shown in Table 3.

**Figure 8:**
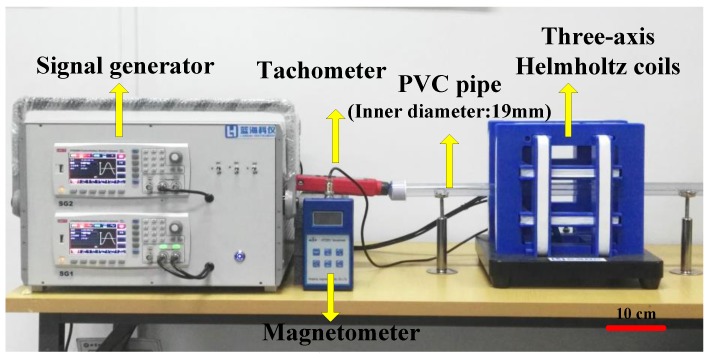
The experimental platform for the active locomotion of the two robots.

Based on the theory mentioned above, an experimental platform was designed to validate the characteristics of multiple capsule robots. The experimental platform, shown in Figure 8, contains signal generators, amplifiers (inside the crate), three-axis Helmholtz coils, a magnetometer (Hengtong, HT201, Shanghai, China), a tachometer (UNI-T, UT372, Dongguan, China), and a polyvinyl chloride (PVC) pipe with the inner diameter of 19 mm.

In our study, the multiple capsule robots performed linear motion in a rigid pipe. The sinusoidal signal output generated from signal generators steered two robots to move forward or backward. The linear movements were controlled by two electric currents. The identical currents flowing in the same pair of coils were set at 3.5 A. The range of frequency was 0 Hz to 20 Hz. Sine signals of each pair of Helmholtz coils with π/2 phase difference produced clockwise rotation and 3π/2 phase difference produced anti-clockwise rotation.

**Figure 9:**
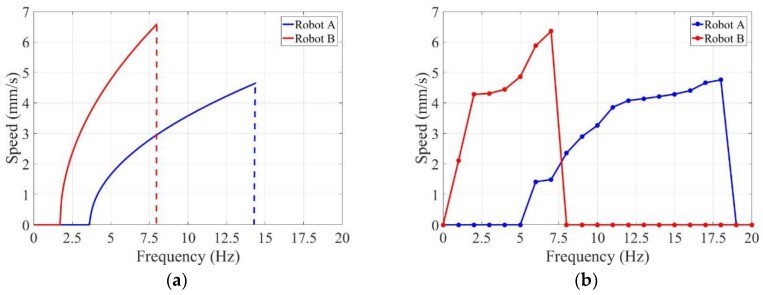
Relationship between axial speeds of individual capsule robots and driven frequencies of rotational electromagnetic field: (**a**) theoretical result; (**b**) experimental result.

The experiments were carried out for Robot A and Robot B separately in the pipe. Each of them were immerged in water. By changing the frequencies of input electric currents, average axial speeds of the two robots were obtained. The theoretical results and experimental results are shown in Figure 9. Both the theoretical results and experimental results revealed that the driven frequencies of two capsule robots had a non-overlapping range. The capsule robots could be controlled independently under an identical rotational electromagnetic field. If we want to steer Robot A, the driven frequencies should be set from 8 Hz to 18 Hz, because in this range Robot B has fallen into a stop. On the contrary, Robot B could be controlled when the driven frequencies were 1 Hz to 5 Hz. The driven frequencies only overlapped at 6 Hz and 7 Hz, and should be discarded. In general, the frequency characteristics may satisfy the experimental requirements in the later part.

As shown in Figure 9a, Robot B was easier to start moving at lower rotational frequencies compared with Robot A. And Robot B tended to fall into a stop at lower rotational frequencies as well. The experimental results demonstrated that the start and step-out frequencies of Robot B were lower than those of Robot A. The start frequency of Robot A was 6 Hz, before which the resistance was larger than axial propulsive force at the low rotational frequencies. Robot B was able to move forward at 1 Hz driven frequency owing to its screw structure which could produce greater propulsion. Moreover, the step-out frequencies of the two robots were 19 Hz and 7 Hz, separately. Higher than these frequencies, the axial speeds began to drop, which means the rotation motion could not keep pace with rotational electromagnetic field.

In addition, the two capsule robots accelerated with the increase in rotational frequencies, because propulsive force increased as the rotational frequencies increased. Figure 9b shows that the axial speeds increased with the frequencies before the maximum at 18 Hz for Robot A and 7 Hz for Robot B. The maximal axial speeds are 4.75 mm/s and 6.35 mm/s, separately. After that, the axial speeds dropped sharply to zero. 

There were errors between the theoretical results and experimental results. Though the correction factor ε was introduced, vibration during rotation, collision with the pipe, and other practical factors were so complex that they could not be characterized by a linear relationship.

### 4.2. Different Motions of the Two Robots

**Figure 10:**
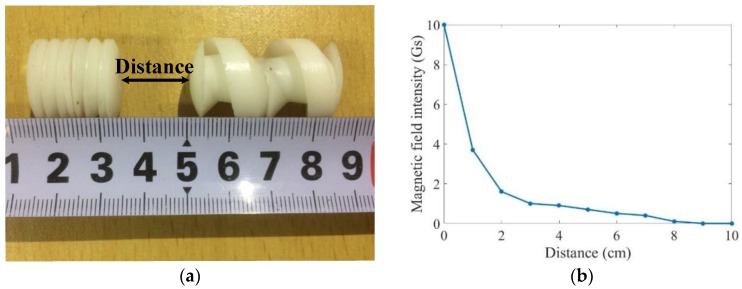
Magnetic field intensity generated by JPMs: (**a**) distance between two JPMs; (**b**) relationship between magnetic field intensity and distance.

Prior to the cooperative locomotion experiments, the magnetic field intensity at different distance intervals was measured by a magnetometer. The magnetic field intensity could be converted to a magnetic field force, which revealed the interaction force during docking and release. In our study, magnetic field intensity depended on the distance of JPMs at the two ends of the robots. The distance is marked in Figure 10a. As shown in Figure 10b, the magnetic field intensity abated as the distance increased. It means two robots were easier to separate at a long distance and stick together when they get closer to each other.

**Figure 11:**
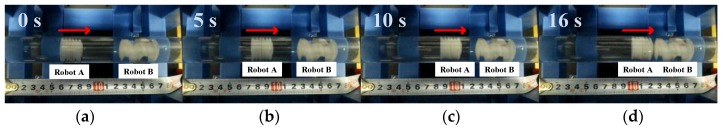
The video snapshots of the docking procedure: (**a**) Robot A is away from Robot B; (**b**) Robot A gets close to Robot B; (**c**) Robot A gets closer to Robot B; and (**d**) the two robots dock together.

To verify the active locomotion in group, the two capsule robots were fed into the same pipe. The motion capabilities of the robots were demonstrated by docking and release procedures in a given position. The video of the navigation in Figure 11 demonstrates the procedure of docking.

The docking procedure can be described as follows. Two capsule robots were located with a separation distance initially. Afterwards, Robot A moved toward Robot B at a certain axial speed. As it got closer to Robot B, the magnetic force of the JPMs pulled them together. The driven frequency of this docking procedure was 8 Hz. Under this driven frequency, Robot A moved forward while Robot B fell into a stop. A series of experiments under different frequencies were set to repeat the procedure. As expected, the time of this procedure decreased as the driven frequencies increased before the step-out frequency of Robot A.

**Figure 12:**
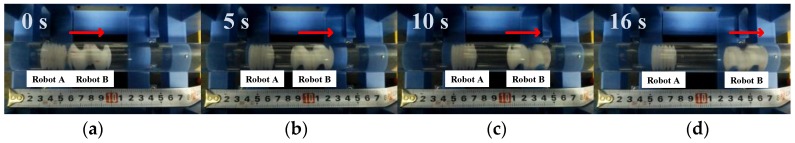
The video snapshots of release procedure: (**a**) two robots dock together; (**b**) Robot B releases from Robot A; (**c**) Robot B moves away from Robot A; and (**d**) Robot B is far away from Robot A.

In the release phase, Robot B rotated under a lower frequency which could not drive Robot A to move forward actively. Overcoming the magnetic force of the JPMs, Robot B moved ahead and the distance between the two capsule robots increased. Ultimately, the two capsule robots released from each other. The driven frequency was set at 3 Hz. Figure 12 demonstrates the procedure of release. In addition, Robot A came out of its initial position. That was because the attractive force of the JPMs also had an effect on Robot A, and the magnetic force turns weakened when the distance of the two capsule robots increased.

**Figure 13:**
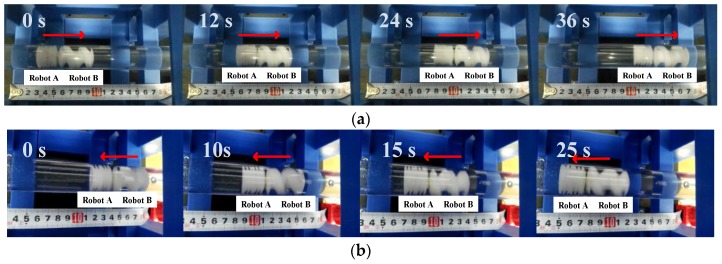
The video snapshots of cooperative locomotion: (**a**) forward locomotion of the whole; (**b**) backward locomotion of the whole.

After docking, the two robots could move together as a whole. Once the hinder robot has higher axial speed, it could push the whole ahead. One of the cooperative locomotion results is shown in Figure 13. When Robot A pushed Robot B, the whole moved forward. They performed backward locomotion when Robot B pushed A. The two robots moved forward together when the driven frequency was set at 11 Hz and the phase difference was π/2. Furthermore, they realized backward movement when the driven frequency was set at 5 Hz and the phase difference was 3π/2.

**Figure 14:**
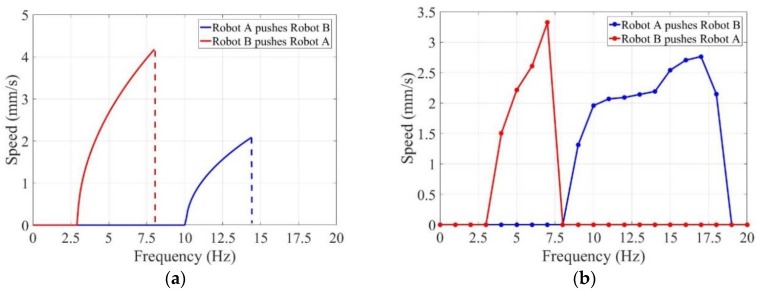
Relationship between axial speeds of the whole and driven frequencies of the rotational electromagnetic field: (**a**) theoretical results; (**b**) experimental results.

In addition, a series of experiments under different driven frequencies were carried out to measure the axial speeds of the whole. The theoretical result and experimental result are shown in Figure 14. Both the theoretical result and experimental result verify the validity of cooperative locomotion of the multiple capsule robots. Evidently the common speeds are much lower if one robot pushes the other. 

As shown in Figure 14a, Robot B pushed Robot A at lower rotational speeds. If the rotational frequencies increased to a certain value, Robot B was pushed by Robot A. The start frequency of the whole was higher than the respective start frequencies of the two capsule robots. The results demonstrated that when the frequency reached a certain value, the whole could move together. The two capsule robots required greater propulsion to overcome resistance of the two capsule robots. The whole can obtain higher axial speeds when Robot B pushed Robot A; it was easy to draw the conclusion that Robot B generated larger propulsion compared with Robot A. As shown in Figure 14b, the practical start frequencies of the whole were 8 Hz when Robot A pushed Robot B and 4 Hz when Robot B pushed Robot A. The maximal axial speed of the whole was 3.32 mm/s.

The errors between the theoretical results and experimental results introduced by vibration during rotation, collision with the pipe, and the interaction of multiple robots exist because a linear relationship is not enough to characterize these complex factors.

## 5. Conclusions

In this paper, a different-frequency driven approach for the millimeter-level multiple capsule robots actuated by identical electromagnetic field is proposed. Steered by the driven frequencies in non-overlapping range, the two robots mimic the independent and cooperative locomotion in narrow tissues of the GI tract or other cavities of the human body. A dynamic model of the robots was established and the multiple start and step-out frequencies were proposed to provide the manipulation guidelines. Different critical driven frequencies can be obtained by designing the geometrical parameters of screw structure and the overlap angles of magnetic polarities. Experimental results of different motions such as docking, release, and cooperative locomotion verified the feasibility of controlling multiple capsule robots with different driven frequencies. 

The start frequencies of the two robots had the interval of 5 Hz and the step-out frequencies were 19 Hz and 8 Hz, separately. The axial speeds of the individual robots peaked at 6.35 mm/s and 4.21 mm/s, respectively. The maximum of cooperative locomotion speed achieved was 3.32 mm/s. The axial speed was lower than the existing rotational robots in the laminar regime, because some of the overlapped frequencies may be discarded to keep only one robot moving. Furthermore, the ranges of driven frequencies were less than 20 Hz, which means the robots were easy to fall into a stop. These results provide significant insights for the development of multiple capsule robots for clinical applications in narrow body cavities.

In real world applications, multiple robots will break the size limitation of individual capsule robots and the space constraint of body cavities. These capsule robots can be swallowed in sequence and form new structures inside the body via docking. Multiple capsule robots with different sensors, drugs, and appliances may be competent for complex treatments such as minimally invasive surgery, and surgeons can customize treatment with different kinds of robots.

Since the dynamic model strongly relies on friction and drag force, our future work will involve collecting information of the GI tract. Besides, various structures for individual capsule robots will be designed to load different sensors, drugs, and appliances. These multiple capsule robots will be guided to form complex structure in three-dimensional space, then in vivo experiments will be conducted in porcine intestine.

## Figures and Tables

**Table 1:** Parameters of the two capsule robots.

Property	Robot A	Robot B
Length of the Body	18 mm	34 mm
Diameter of the Body	16 mm	16 mm
Weight	3.64 g	5.59 g
Radius of the DPMs	3 mm	3 mm
Radius of the JPMs	1 mm	1 mm

**Table 2:** Specifications of the three-axis Helmholtz coils.

Property	X-axis	Y-axis	Z-axis
Length × Width	18 cm × 18 cm	22 cm × 22 cm	26 cm × 26cm
Turns	500	620	740
Magnetic Field	3880.9 (A/m)	3880.9 (A/m)	3880.9 (A/m)
Materials	copper	copper	copper
Diameter of Copper Wires	1.25 mm	1.25 mm	1.25 mm

**Table 3:** Special design of the two capsule robots.

Property	Robot A	Robot B
Pitch (λ)	3 mm	12 mm
Spiral Numbers (n)	4	2
Blade Angle (θ)	π/4	π/4
Lead Angle (δ)	π/18	π/6
Depth of the Screws (H)	2 mm	4 mm
Width of the Screws (a)	2 mm	4 mm
Quantity of DPMs	1	2
Overlap Angle of Magnetic Polarities (Φ)	-	π/4
